# Crystal structure of {bis­[(1*H*-benzimid­azol-2-yl-κ*N* ^3^)meth­yl]sulfane}dichloridomercury(II)

**DOI:** 10.1107/S205698901502349X

**Published:** 2015-12-12

**Authors:** Mehdi Bouchouit, Saida Benzerka, Abdelmalek Bouraiou, Hocine Merazig, Ali Belfaitah, Sofiane Bouacida

**Affiliations:** a Unité de Recherche de Chimie de l’Environnement et Moléculaire Structurale CHEMS, Université Frères Montouri, Constantine 25000, Algeria; b Laboratoire de Synthèse des Molécules d’Intérêts Biologiques, Université des Frères Mentouri, Constantine 25000, Algeria; c Laboratoire des Produits Naturels d’Origine Végétale et de Synthèse Organique, PHYSYNOR, Université Frères Montouri, Constantine 25000, Algeria; d Département Sciences de la Matière, Faculté des Sciences Exactes et Sciences de la Nature et de la Vie, Université Oum El Bouaghi, Algeria

**Keywords:** crystal structure, benzimidazole derivatives, mercury(II), hydrogen-bond patterns

## Abstract

In the asymmetric unit of the title compound, [HgCl_2_(C_16_H_14_N_4_S)], the Hg^II^ cation is linked to two Cl atoms and two imidazole N atoms of the chelating bis­[(1*H*-benzimidazol-2-yl)meth­yl]sulfane ligand, forming a slightly distorted tetra­hedral environment. The substitued imidazole rings of the ligand are almost perfectly planar [with maximum deviations of 0.017 (3) and 0.012 (3) Å] and form a dihedral angle of 42.51 (5)°. The crystal packing can be described as alternating layers parallel to (010). In this arrangement, N—H⋯Cl hydrogen bonds between the N—H groups of the benzimidazole moieties and chloride ligands are responsible for the formation of the chain-like packing pattern along [010] exhibiting a *C*(6) graph-set motif.

## Related literature   

For the synthesis and applications of benzimiazole derivatives, see: Tiwari *et al.* (2007 ▸); Gowda *et al.* (2009 ▸); Sondhi *et al.*, (2010 ▸). For the coordination of benzimiazole derivatives, see: Téllez *et al.* (2008 ▸); Sundberg & Martin (1974 ▸); Reedijk (1987 ▸).
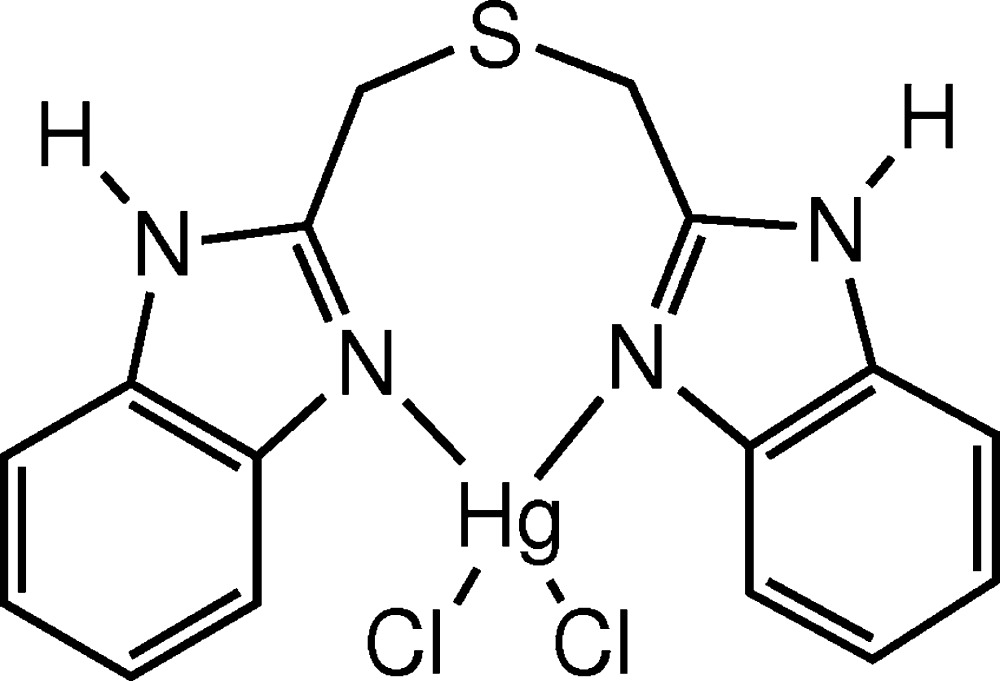


## Experimental   

### Crystal data  

[HgCl_2_(C_16_H_14_N_4_S)]
*M* *_r_* = 565.86
Orthorhombic, 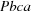

*a* = 13.8558 (3) Å
*b* = 15.4983 (4) Å
*c* = 16.1108 (4) Å
*V* = 3459.66 (14) Å^3^
*Z* = 8
Mo *K*α radiation
μ = 9.33 mm^−1^
*T* = 295 K
0.16 × 0.11 × 0.09 mm

### Data collection  

Bruker APEXII diffractometer
Absorption correction: multi-scan (*SADABS*; Sheldrick, 2002 ▸) *T*_min_ = 0.646, *T*_max_ = 0.746
78309 measured reflections
5594 independent reflections
4351 reflections with *I* > 2σ(*I*)
*R*_int_ = 0.035

### Refinement  

*R*[*F* ^2^ > 2σ(*F* ^2^)] = 0.020
*wR*(*F* ^2^) = 0.046
*S* = 1.01
5594 reflections
217 parameters
H-atom parameters constrained
Δρ_max_ = 0.86 e Å^−3^
Δρ_min_ = −0.93 e Å^−3^

### 

Data collection: *APEX2* (Bruker, 2011 ▸); cell refinement: *SAINT* (Bruker, 2011 ▸); data reduction: *SAINT*; program(s) used to solve structure: *SIR2002* (Burla *et al.*, 2005 ▸); program(s) used to refine structure: *SHELXT* (Sheldrick, 2015 ▸); molecular graphics: *ORTEP-3 for Windows* (Farrugia, 2012 ▸) and *DIAMOND* (Brandenburg & Berndt, 2001 ▸); software used to prepare material for publication: *WinGX* (Farrugia, 2012 ▸) and *CRYSCAL* (T. Roisnel, local program).

## Supplementary Material

Crystal structure: contains datablock(s) I. DOI: 10.1107/S205698901502349X/im2475sup1.cif

Structure factors: contains datablock(s) I. DOI: 10.1107/S205698901502349X/im2475Isup2.hkl

**Fig. 1.**
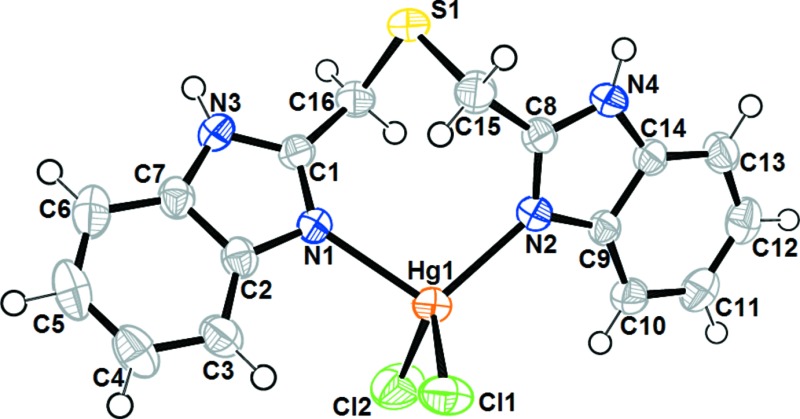
The molecular structure of the title compound with the atomic labelling scheme. Displacement ellipsoids are drawn at the 50% probability level. H atoms are represented as small spheres of arbitrary radii.

**Fig. 2.**
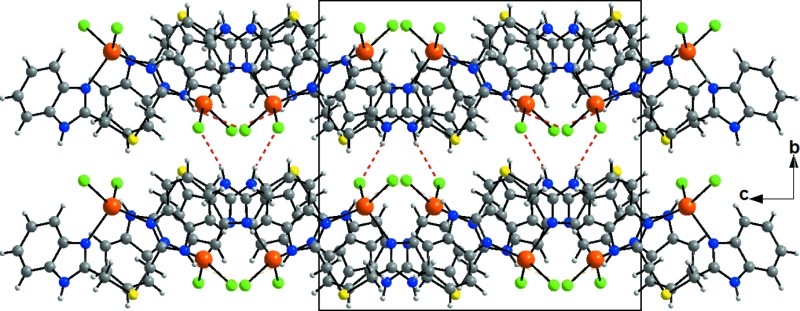
Alternating layers parallel to (010) plane of (I) at b = 1/4 and b = 3/4, viewed down the a axis.

. DOI: 10.1107/S205698901502349X/im2475fig1.tif
The mol­ecular structure of the title compound with the atomic labelling scheme. Displacement ellipsoids are drawn at the 50% probability level. H atoms are represented as small spheres of arbitrary radii.

b b a . DOI: 10.1107/S205698901502349X/im2475fig2.tif
Alternating layers parallel to (010) plane of (I) at *b* = 1/4 and *b* = 3/4, viewed down the *a* axis.

CCDC reference: 1440754

Additional supporting information:  crystallographic information; 3D view; checkCIF report

## Figures and Tables

**Table 1:** Hydrogen-bond geometry (Å, °)

*D*—H⋯*A*	*D*—H	H⋯*A*	*D*⋯*A*	*D*—H⋯*A*
N3—H3*N*⋯Cl1^i^	0.86	2.35	3.178 (2)	163
N4—H4*N*⋯Cl2^ii^	0.86	2.76	3.508 (2)	147

Symmetry codes: (i) 
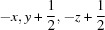
; (ii) 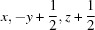.

## supplementary crystallographic information

### S1. Chemical context

Benzimidazole derivatives are reported to be physiologically and pharmacologically active (Tiwari, *et al.*, 2007) and have shown different therapeutic properties such as anti­hypertensive, anti­coagulant, anti­allergic, analgesic, anti-inflammatory, anti­microbial, anti­parasitic and anti­oxidant (Thimme Gowda, *et al.*, 2009). Because of their significant medicinal importance, the synthesis of substituted benzimidazoles has become a focus of synthetic organic chemistry (Sondhi, *et al.*, 2010). Benzimidazoles act as good ligands towards transition metal ions and give place to a variety of metal-ligand coordination modes. Their reactions with metal salts have played a significant role in the development of coordination chemistry of this class of ligands (Téllez, *et al.*, 2008). Several research groups have investigated the coordination behavior of benzimidazole derivatives towards transition metal ions (Sundberg & Martin 1974; Reedijk, 1987) and numerous studies concerned with the biological activity of coordination compounds containing benzimidazole derivatives are also in progress.

### S2. Structural commentary

Herein, we report the synthesis and structure determination of a new complex based on mercury and a chelating bis-benzimidazole ligand. The molecular structure of (I) together with the atomic numbering scheme is illustrated in Fig. 1.

In the asymmetric unit of [HgCl_2_(C_16_H_14_N_4_S)], (I), the Hg^II^ cation is linked to two chlorine atoms and two imidazole N atoms of the chelating ligand bis­((1*H*-benzo[*d*]imidazol-2-yl)methyl)­sulfane forming a slightly distorted tetra­hedral environment [Hg—N = 2.2991 (19) and 2.2471 (19) Å; Hg—Cl = 2.4459 (7) and 2.4554 (7) Å].

Substitued imidazole rings of the ligand are almost perfectly planar [with maximum deviation of 0.0170 (26) Å at C13 and 0.0121 (28) Å at C5] and form a dihedral angle of 42.51 (5)°.

### S3. Supra­molecular features

The crystal packing can be described by alternating layers parallel to (010) (Figure 2). In this arragement N—H···Cl hydrogen bonds between amine moities and chloride ligands are responsible for the formation of the one-dimensional chain-like packing pattern exhibiting a C_1_^1^(6) graph set motif (Etter *et al.*, 1990; Bernstein *et al.*, 1995). Additional hydrogen-bonding parameters are listed in Table 1. The packing is consolidated by π-π stacking inter­actions with centroid to centroid distances of 3.5525 (14) to 3.6963 (14) Å between benzimidazole rings. These inter­actions link the molecules within the layers and also link the layers together reinforcing the cohesion of the complex structure.

### S4. Synthesis and crystallization

Complex I was prepared by stirring 294 mg (1 mmol) of bis­((1*H*-benzo[*d*]imidazol-2-yl)methyl)­sulfane and 271 mg (1 mmol) HgCl_2_ in 20 ml of methanol for 24 h. The obtained solid was filtered and recrystallized by diffusion of di­ethyl ether into a DMF solution of the title compound at room temperature. Colorless crystals (yield: 83%) suitable for the X-ray diffraction study were obtained by this procedure.

### S5. Refinement

Crystal data, data collection and structure refinement details are summarized in Table 1. H atoms were localized on Fourier maps but introduced into calculated positions and treated as riding on their parent atom (C or N) with C—H = 0.93 Å (aromatic), C—H = 0.97 Å (methyl­ene) and N—H = 0.86 Å (amine) with *U*_iso_(H) = 1.2*U*_eq_ (C or N).

### Crystal data

**Table:**

[HgCl_2_(C_16_H_14_N_4_S)]	*D*_x_ = 2.173 Mg m^−^^3^
*M**_r_* = 565.86	Mo *K*α radiation, λ = 0.71073 Å
Orthorhombic, *P**b**c**a*	Cell parameters from 9958 reflections
*a* = 13.8558 (3) Å	θ = 2.3–29.6°
*b* = 15.4983 (4) Å	µ = 9.33 mm^−^^1^
*c* = 16.1108 (4) Å	*T* = 295 K
*V* = 3459.66 (14) Å^3^	Prism, colorless
*Z* = 8	0.16 × 0.11 × 0.09 mm
*F*(000) = 2144	

### Data collection

**Table:**

Bruker APEXII diffractometer	5594 independent reflections
Radiation source: Enraf Nonius FR590	4351 reflections with *I* > 2σ(*I*)
Graphite monochromator	*R*_int_ = 0.035
CCD rotation images, thick slices scans	θ_max_ = 31.2°, θ_min_ = 2.5°
Absorption correction: multi-scan (*SADABS*; Sheldrick, 2002)	*h* = −20→20
*T*_min_ = 0.646, *T*_max_ = 0.746	*k* = −22→22
78309 measured reflections	*l* = −22→23

### Refinement

**Table:**

Refinement on *F*^2^	Primary atom site location: structure-invariant direct methods
Least-squares matrix: full	Secondary atom site location: difference Fourier map
*R*[*F*^2^ > 2σ(*F*^2^)] = 0.020	Hydrogen site location: inferred from neighbouring sites
*wR*(*F*^2^) = 0.046	H-atom parameters constrained
*S* = 1.01	*w* = 1/[σ^2^(*F*_o_^2^) + (0.0162*P*)^2^ + 4.119*P*] where *P* = (*F*_o_^2^ + 2*F*_c_^2^)/3
5594 reflections	(Δ/σ)_max_ = 0.001
217 parameters	Δρ_max_ = 0.86 e Å^−^^3^
0 restraints	Δρ_min_ = −0.93 e Å^−^^3^

### Special details

**Table:**

Geometry. All e.s.d.'s (except the e.s.d. in the dihedral angle between two l.s. planes) are estimated using the full covariance matrix. The cell e.s.d.'s are taken into account individually in the estimation of e.s.d.'s in distances, angles and torsion angles; correlations between e.s.d.'s in cell parameters are only used when they are defined by crystal symmetry. An approximate (isotropic) treatment of cell e.s.d.'s is used for estimating e.s.d.'s involving l.s. planes.
Refinement. Refinement of *F*^2^ against ALL reflections. The weighted *R*-factor *wR* and goodness of fit *S* are based on *F*^2^, conventional *R*-factors *R* are based on *F*, with *F* set to zero for negative *F*^2^. The threshold expression of *F*^2^ > σ(*F*^2^) is used only for calculating *R*-factors(gt) *etc*. and is not relevant to the choice of reflections for refinement. *R*-factors based on *F*^2^ are statistically about twice as large as those based on *F*, and *R*- factors based on ALL data will be even larger.

### Fractional atomic coordinates and isotropic or equivalent isotropic displacement parameters (Å^2^)

**Table:**

	*x*	*y*	*z*	*U*_iso_*/*U*_eq_	
C1	−0.02521 (16)	0.28013 (15)	0.20752 (14)	0.0275 (5)	
C2	−0.08439 (18)	0.21383 (18)	0.17932 (16)	0.0350 (5)	
H2	−0.0882	0.1613	0.207	0.042*	
C3	−0.13721 (19)	0.2296 (2)	0.10828 (17)	0.0425 (7)	
H3	−0.1779	0.1868	0.088	0.051*	
C4	−0.1311 (2)	0.3079 (2)	0.06612 (17)	0.0447 (7)	
H4	−0.1673	0.3155	0.0181	0.054*	
C5	−0.07323 (19)	0.3743 (2)	0.09337 (17)	0.0404 (6)	
H5	−0.0696	0.4266	0.0652	0.048*	
C6	−0.02053 (17)	0.35897 (16)	0.16529 (15)	0.0289 (5)	
C7	0.07692 (16)	0.36125 (15)	0.27412 (15)	0.0272 (5)	
C8	0.14891 (18)	0.39298 (16)	0.33483 (16)	0.0332 (5)	
H8A	0.193	0.4319	0.3068	0.04*	
H8B	0.1862	0.3444	0.3551	0.04*	
C9	0.0330 (2)	0.36181 (17)	0.47575 (17)	0.0355 (5)	
H9A	−0.0062	0.3861	0.5198	0.043*	
H9B	−0.0101	0.3339	0.4366	0.043*	
C10	0.09852 (17)	0.29578 (15)	0.51189 (14)	0.0272 (4)	
C11	0.17866 (16)	0.17664 (14)	0.53263 (14)	0.0247 (4)	
C12	0.21845 (18)	0.09428 (16)	0.52899 (16)	0.0321 (5)	
H12	0.2047	0.0568	0.4855	0.039*	
C13	0.27933 (19)	0.07089 (18)	0.59296 (17)	0.0388 (6)	
H13	0.3068	0.0161	0.5925	0.047*	
C14	0.30100 (19)	0.12684 (19)	0.65845 (17)	0.0394 (6)	
H14	0.3426	0.1085	0.7001	0.047*	
C15	0.26224 (19)	0.20829 (18)	0.66267 (16)	0.0362 (6)	
H15	0.277	0.2458	0.7059	0.043*	
C16	0.19957 (16)	0.23173 (15)	0.59886 (14)	0.0274 (5)	
N1	0.03651 (14)	0.28386 (12)	0.27585 (12)	0.0272 (4)	
N2	0.11618 (14)	0.21920 (12)	0.47882 (12)	0.0268 (4)	
N3	0.04426 (15)	0.40839 (13)	0.20965 (13)	0.0311 (4)	
H3N	0.061	0.4605	0.1981	0.037*	
N4	0.14764 (15)	0.30614 (13)	0.58354 (12)	0.0310 (4)	
H4N	0.1468	0.3514	0.6144	0.037*	
S1	0.09600 (6)	0.44882 (4)	0.42297 (4)	0.04249 (16)	
Cl1	−0.09138 (5)	0.08943 (4)	0.37366 (4)	0.04179 (15)	
Cl2	0.17398 (6)	0.08112 (5)	0.27101 (5)	0.0583 (2)	
Hg1	0.063397 (7)	0.164790 (6)	0.357762 (6)	0.03372 (3)	

### Atomic displacement parameters (Å^2^)

**Table:**

	*U*^11^	*U*^22^	*U*^33^	*U*^12^	*U*^13^	*U*^23^
C1	0.0263 (10)	0.0325 (12)	0.0237 (11)	0.0028 (9)	0.0011 (9)	−0.0008 (9)
C2	0.0365 (13)	0.0382 (14)	0.0305 (13)	−0.0066 (10)	−0.0014 (10)	−0.0039 (11)
C3	0.0328 (13)	0.0611 (19)	0.0336 (14)	−0.0083 (12)	−0.0045 (11)	−0.0068 (13)
C4	0.0345 (13)	0.072 (2)	0.0279 (13)	0.0039 (13)	−0.0069 (11)	0.0007 (13)
C5	0.0421 (14)	0.0491 (16)	0.0301 (13)	0.0088 (12)	0.0002 (11)	0.0075 (12)
C6	0.0299 (11)	0.0332 (12)	0.0236 (11)	0.0046 (9)	0.0023 (9)	0.0005 (9)
C7	0.0309 (11)	0.0238 (10)	0.0268 (11)	0.0003 (8)	0.0015 (9)	0.0001 (9)
C8	0.0380 (12)	0.0284 (12)	0.0331 (13)	−0.0074 (10)	−0.0037 (10)	−0.0004 (10)
C9	0.0421 (13)	0.0332 (12)	0.0311 (13)	0.0104 (11)	0.0032 (11)	−0.0015 (10)
C10	0.0309 (11)	0.0270 (11)	0.0238 (11)	0.0008 (9)	0.0030 (9)	−0.0001 (9)
C11	0.0265 (10)	0.0259 (11)	0.0216 (10)	−0.0032 (8)	0.0019 (8)	0.0019 (8)
C12	0.0374 (12)	0.0281 (11)	0.0309 (13)	0.0009 (10)	0.0024 (10)	−0.0010 (10)
C13	0.0382 (13)	0.0357 (13)	0.0426 (15)	0.0062 (11)	0.0017 (11)	0.0098 (11)
C14	0.0355 (13)	0.0461 (15)	0.0366 (15)	−0.0004 (12)	−0.0050 (11)	0.0117 (12)
C15	0.0407 (14)	0.0405 (14)	0.0274 (12)	−0.0082 (11)	−0.0055 (10)	0.0009 (10)
C16	0.0299 (11)	0.0272 (11)	0.0250 (11)	−0.0042 (9)	0.0020 (9)	0.0019 (9)
N1	0.0315 (9)	0.0242 (9)	0.0259 (10)	−0.0014 (7)	−0.0034 (8)	0.0016 (8)
N2	0.0299 (9)	0.0253 (9)	0.0252 (10)	−0.0001 (7)	0.0000 (8)	−0.0015 (8)
N3	0.0395 (11)	0.0247 (9)	0.0291 (10)	−0.0005 (8)	−0.0004 (8)	0.0045 (8)
N4	0.0426 (11)	0.0249 (9)	0.0254 (10)	−0.0004 (8)	0.0015 (9)	−0.0043 (8)
S1	0.0708 (5)	0.0221 (3)	0.0345 (3)	0.0013 (3)	−0.0047 (3)	−0.0040 (3)
Cl1	0.0454 (3)	0.0340 (3)	0.0460 (4)	−0.0095 (3)	0.0061 (3)	−0.0095 (3)
Cl2	0.0735 (5)	0.0413 (4)	0.0601 (5)	0.0137 (4)	0.0196 (4)	−0.0056 (3)
Hg1	0.04398 (6)	0.02599 (5)	0.03120 (5)	−0.00193 (4)	−0.00583 (4)	−0.00131 (4)

### Geometric parameters (Å, º)

**Table:**

C1—C2	1.391 (3)	C9—H9B	0.97
C1—N1	1.395 (3)	C10—N2	1.324 (3)
C1—C6	1.400 (3)	C10—N4	1.350 (3)
C2—C3	1.380 (4)	C11—N2	1.391 (3)
C2—H2	0.93	C11—C12	1.392 (3)
C3—C4	1.393 (4)	C11—C16	1.397 (3)
C3—H3	0.93	C12—C13	1.380 (4)
C4—C5	1.378 (4)	C12—H12	0.93
C4—H4	0.93	C13—C14	1.398 (4)
C5—C6	1.390 (4)	C13—H13	0.93
C5—H5	0.93	C14—C15	1.373 (4)
C6—N3	1.380 (3)	C14—H14	0.93
C7—N1	1.324 (3)	C15—C16	1.394 (3)
C7—N3	1.348 (3)	C15—H15	0.93
C7—C8	1.481 (3)	C16—N4	1.382 (3)
C8—S1	1.817 (3)	N1—Hg1	2.2991 (19)
C8—H8A	0.97	N2—Hg1	2.2471 (19)
C8—H8B	0.97	N3—H3N	0.86
C9—C10	1.487 (3)	N4—H4N	0.86
C9—S1	1.818 (3)	Cl1—Hg1	2.4554 (7)
C9—H9A	0.97	Cl2—Hg1	2.4459 (7)
			
C2—C1—N1	130.5 (2)	N2—C11—C16	108.38 (19)
C2—C1—C6	120.9 (2)	C12—C11—C16	120.7 (2)
N1—C1—C6	108.6 (2)	C13—C12—C11	116.8 (2)
C3—C2—C1	116.9 (3)	C13—C12—H12	121.6
C3—C2—H2	121.5	C11—C12—H12	121.6
C1—C2—H2	121.5	C12—C13—C14	122.1 (3)
C2—C3—C4	121.8 (3)	C12—C13—H13	118.9
C2—C3—H3	119.1	C14—C13—H13	118.9
C4—C3—H3	119.1	C15—C14—C13	121.6 (2)
C5—C4—C3	122.1 (3)	C15—C14—H14	119.2
C5—C4—H4	119	C13—C14—H14	119.2
C3—C4—H4	119	C14—C15—C16	116.5 (2)
C4—C5—C6	116.3 (3)	C14—C15—H15	121.7
C4—C5—H5	121.8	C16—C15—H15	121.7
C6—C5—H5	121.8	N4—C16—C15	132.4 (2)
N3—C6—C5	132.7 (2)	N4—C16—C11	105.41 (19)
N3—C6—C1	105.2 (2)	C15—C16—C11	122.2 (2)
C5—C6—C1	122.0 (2)	C7—N1—C1	106.27 (19)
N1—C7—N3	111.4 (2)	C7—N1—Hg1	132.12 (16)
N1—C7—C8	124.9 (2)	C1—N1—Hg1	121.26 (15)
N3—C7—C8	123.7 (2)	C10—N2—C11	106.81 (19)
C7—C8—S1	113.73 (18)	C10—N2—Hg1	128.73 (16)
C7—C8—H8A	108.8	C11—N2—Hg1	124.43 (14)
S1—C8—H8A	108.8	C7—N3—C6	108.5 (2)
C7—C8—H8B	108.8	C7—N3—H3N	125.8
S1—C8—H8B	108.8	C6—N3—H3N	125.8
H8A—C8—H8B	107.7	C10—N4—C16	108.43 (19)
C10—C9—S1	113.62 (19)	C10—N4—H4N	125.8
C10—C9—H9A	108.8	C16—N4—H4N	125.8
S1—C9—H9A	108.8	C8—S1—C9	101.89 (12)
C10—C9—H9B	108.8	N2—Hg1—N1	104.46 (7)
S1—C9—H9B	108.8	N2—Hg1—Cl2	119.40 (5)
H9A—C9—H9B	107.7	N1—Hg1—Cl2	101.48 (5)
N2—C10—N4	111.0 (2)	N2—Hg1—Cl1	111.85 (5)
N2—C10—C9	124.9 (2)	N1—Hg1—Cl1	107.47 (5)
N4—C10—C9	124.1 (2)	Cl2—Hg1—Cl1	110.77 (3)
N2—C11—C12	130.9 (2)		
			
N1—C1—C2—C3	−179.5 (2)	C2—C1—N1—Hg1	5.2 (3)
C6—C1—C2—C3	0.3 (4)	C6—C1—N1—Hg1	−174.53 (15)
C1—C2—C3—C4	0.6 (4)	N4—C10—N2—C11	1.2 (3)
C2—C3—C4—C5	−0.9 (5)	C9—C10—N2—C11	−178.4 (2)
C3—C4—C5—C6	0.3 (4)	N4—C10—N2—Hg1	−176.69 (15)
C4—C5—C6—N3	179.2 (3)	C9—C10—N2—Hg1	3.7 (3)
C4—C5—C6—C1	0.5 (4)	C12—C11—N2—C10	178.5 (2)
C2—C1—C6—N3	−179.8 (2)	C16—C11—N2—C10	−1.2 (2)
N1—C1—C6—N3	0.0 (3)	C12—C11—N2—Hg1	−3.4 (3)
C2—C1—C6—C5	−0.8 (4)	C16—C11—N2—Hg1	176.80 (14)
N1—C1—C6—C5	178.9 (2)	N1—C7—N3—C6	−0.9 (3)
N1—C7—C8—S1	−91.1 (3)	C8—C7—N3—C6	179.1 (2)
N3—C7—C8—S1	88.9 (3)	C5—C6—N3—C7	−178.3 (3)
S1—C9—C10—N2	−102.2 (3)	C1—C6—N3—C7	0.6 (3)
S1—C9—C10—N4	78.2 (3)	N2—C10—N4—C16	−0.8 (3)
N2—C11—C12—C13	179.7 (2)	C9—C10—N4—C16	178.9 (2)
C16—C11—C12—C13	−0.6 (3)	C15—C16—N4—C10	179.2 (3)
C11—C12—C13—C14	−0.3 (4)	C11—C16—N4—C10	0.0 (3)
C12—C13—C14—C15	0.3 (4)	C7—C8—S1—C9	67.4 (2)
C13—C14—C15—C16	0.7 (4)	C10—C9—S1—C8	67.0 (2)
C14—C15—C16—N4	179.2 (2)	C10—N2—Hg1—N1	26.7 (2)
C14—C15—C16—C11	−1.6 (4)	C11—N2—Hg1—N1	−150.88 (17)
N2—C11—C16—N4	0.8 (2)	C10—N2—Hg1—Cl2	139.14 (18)
C12—C11—C16—N4	−179.0 (2)	C11—N2—Hg1—Cl2	−38.46 (19)
N2—C11—C16—C15	−178.6 (2)	C10—N2—Hg1—Cl1	−89.2 (2)
C12—C11—C16—C15	1.6 (3)	C11—N2—Hg1—Cl1	93.18 (17)
N3—C7—N1—C1	0.9 (3)	C7—N1—Hg1—N2	29.2 (2)
C8—C7—N1—C1	−179.1 (2)	C1—N1—Hg1—N2	−158.52 (16)
N3—C7—N1—Hg1	173.99 (16)	C7—N1—Hg1—Cl2	−95.5 (2)
C8—C7—N1—Hg1	−6.0 (4)	C1—N1—Hg1—Cl2	76.74 (17)
C2—C1—N1—C7	179.2 (3)	C7—N1—Hg1—Cl1	148.2 (2)
C6—C1—N1—C7	−0.5 (3)	C1—N1—Hg1—Cl1	−39.57 (18)

### Hydrogen-bond geometry (Å, º)

**Table:**

*D*—H···*A*	*D*—H	H···*A*	*D*···*A*	*D*—H···*A*
N3—H3*N*···Cl1^i^	0.86	2.35	3.178 (2)	163
N4—H4*N*···Cl2^ii^	0.86	2.76	3.508 (2)	147

Symmetry codes: (i) −*x*, *y*+1/2, −*z*+1/2; (ii) *x*, −*y*+1/2, *z*+1/2.
