# Supplementary figures and images for: Crystal structure of {bis­[(1H-benzimid­azol-2-yl-κN 3)meth­yl]sulfane}dichloridomercury(II)
Source: Acta Crystallogr E Crystallogr Commun. 2015 Dec 12;71(Pt 12):m253–4. doi: 10.1107/S205698901502349X (PMC4719860; doi:10.1107/S205698901502349X)

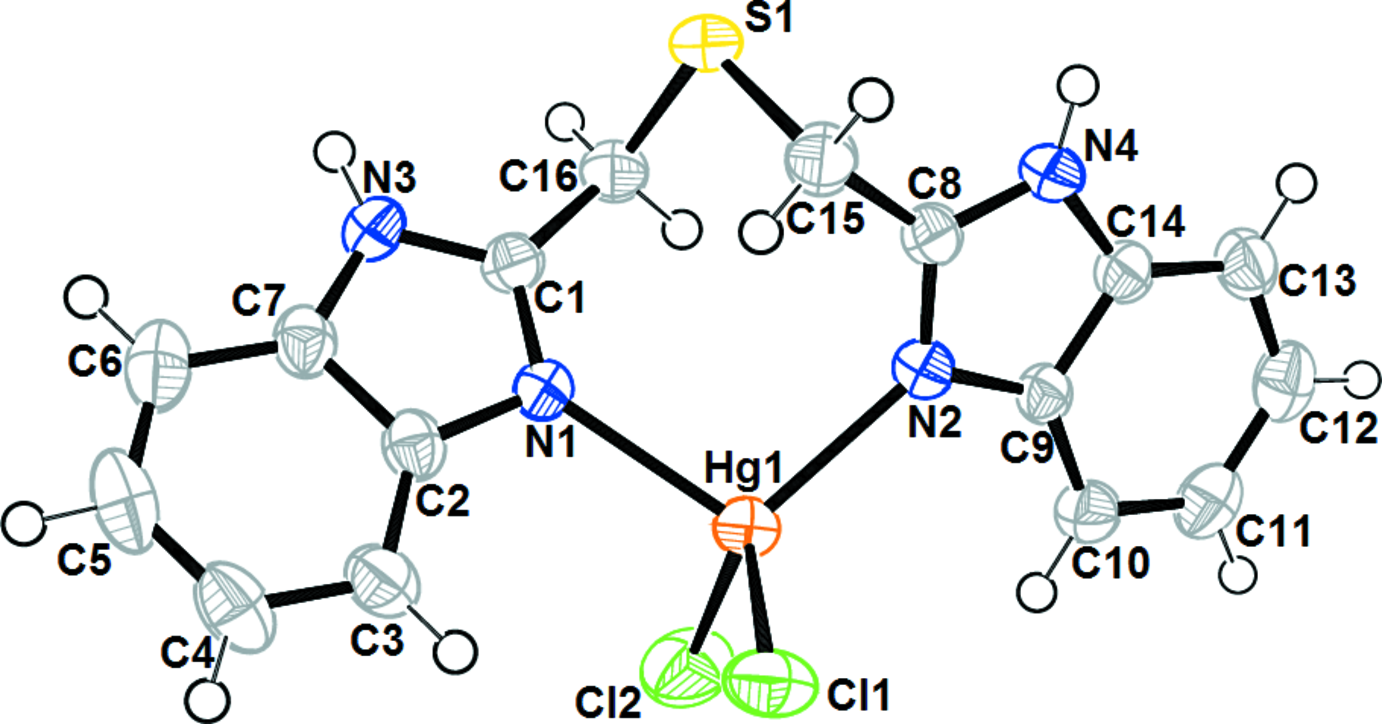

Supplement: Supplementary file 3 [file e-71-0m253-fig1.tif]

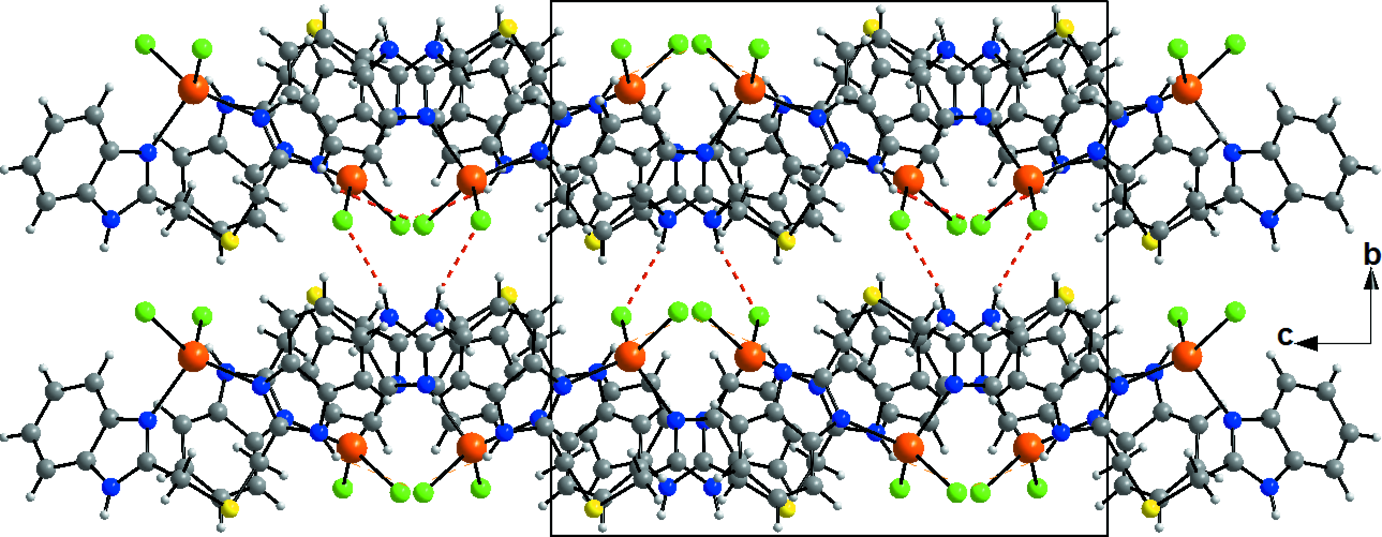

Supplement: Supplementary file 4 [file e-71-0m253-fig2.tif]
